# Structure and Properties of DNA Molecules Over The Full Range of Biologically Relevant Supercoiling States

**DOI:** 10.1038/s41598-018-24499-5

**Published:** 2018-04-18

**Authors:** Paolo Bettotti, Valeria Visone, Lorenzo Lunelli, Giuseppe Perugino, Maria Ciaramella, Anna Valenti

**Affiliations:** 1 Nanoscience Laboratory, Department of Physics, University of Trento, Via Sommarive 14, I-38123 Povo (Trento), Italy; 2 Institute of Biosciences and Bioresources, National Research Council of Italy, Via Pietro Castellino 111, 80131 Napoli, Italy; 3 Laboratory of Biomarker Studies and Structure Analysis for Health, FBK-Fondazione Bruno Kessler, Via Sommarive 18, 38123 Povo, Trento Italy; 4 Institute of Biophysics, National Research Council of Italy, Trento, Italy

## Abstract

Topology affects physical and biological properties of DNA and impacts fundamental cellular processes, such as gene expression, genome replication, chromosome structure and segregation. In all organisms DNA topology is carefully modulated and the supercoiling degree of defined genome regions may change according to physiological and environmental conditions. Elucidation of structural properties of DNA molecules with different topology may thus help to better understand genome functions. Whereas a number of structural studies have been published on highly negatively supercoiled DNA molecules, only preliminary observations of highly positively supercoiled are available, and a description of DNA structural properties over the full range of supercoiling degree is lacking. Atomic Force Microscopy (AFM) is a powerful tool to study DNA structure at single molecule level. We here report a comprehensive analysis by AFM of DNA plasmid molecules with defined supercoiling degree, covering the full spectrum of biologically relevant topologies, under different observation conditions. Our data, supported by statistical and biochemical analyses, revealed striking differences in the behavior of positive and negative plasmid molecules.

## Introduction

DNA topology is an intrinsic property of DNA molecules, and is controlled by the direct action of DNA topoisomerases^1–8^. These are enzymes essential for proliferation and survival of all cells and are indeed important targets for chemotherapeutic drugs. *In vitro*, DNA topoisomerases are able to induce significant changes of linking number (ΔLk; see the Methods Section for definition of the topological parameters) of covalently closed DNA molecules, by either removing or introducing supercoils. Each enzyme has its own specificity, and can modify the ΔLk stepwise, leading to production of a range of differently supercoiled molecules.

*In vivo*, genomic DNA is organized in topologically closed domains, whose supercoiling degree is strictly regulated, given the great impact of topology on all DNA activities^9,10^. In organisms living in the range of mesophilic temperatures, DNA is in general negatively supercoiled (−SC); thermophilic microorganisms, living in environmental niches above 70 °C, possess a special DNA topoisomerase called reverse gyrase (RG), which is able to catalyze positive supercoiling^11–13^. Recent results show that RG is essential for growth at 95 °C of a hyperthermophilic species, suggesting that the presence of the enzyme is necessary to maintain the correct DNA supercoiling in organisms living at high temperatures^14^.

Positively supercoiled (+SC) DNA accumulates in every organism during DNA transactions, such as replication and transcription^15^; moreover, +SC DNA prevents extensive reversal of replication forks in the presence of replication blocking lesions^16^, promotes telomere resolutions^17^ and marks centromeres as unique chromosome loci^18^. In addition, condensin and cohesin complexes, which play essential role in chromosome architecture and segregation during mitosis and meiosis, induce positive supercoiling^19,20^. Although current techniques do not allow determination of supercoiling degree *in vivo* in real time, it is likely that the full range of topological degree may occur during the cell life and DNA supercoiling may vary significantly over the cell cycle, in distinct genome locations as well as in response to environmental stimuli. Thus, understanding how DNA is organized over the full range of supercoiling degree may provide crucial insights into the principles that underlie genomic organization and regulation in living organisms.

So far, the structure of −SC plasmids has been investigated by several techniques^21,22^. Electron (SEM, TEM) and scanning probe microscopy (SPM) are the *de-facto* techniques to visualize DNA at single molecule level and to unveil structural details. All these techniques suffer from possible artifacts during sample preparation, which might influence the measurements outcome^23–26^. Atomic force microscopy (AFM) may in part overcome these limitations, as it allows to control several parameters during plasmids deposition (e.g. buffer ionic strength, temperature, etc.) and to simulate *in-vivo* conditions by performing measurements in liquids. Although many structural studies have been reported on −SC plasmids, little information is available for +SC plasmids. Only recently two papers reported the first observations at single molecule level of +SC DNA molecules, giving important impulse to the field^27,28^. However, further analyses are required to extend these results and fill important gaps. Indeed, Irobalieva *et al*. used cryo-tomography to analyze DNA minicircles of a few hundred base pairs with defined degrees of supercoiling. Although these molecules are useful models, they can only accommodate a few supercoils, due to their short size. More recently, Li *et al*. analyzed by AFM +SC plasmids and measured different lengths between positive and negative topoisomers, whose ΔLk was however undefined. These measurements rely on few molecules only and their conclusions might be of limited relevance because of the reduced ensemble investigated^28^.

In the present work, we present a systematic AFM analysis of DNA plasmids with ΔLk covering a wide range of supercoiling (ranging from −12 to +12). Observations were performed under a number of different conditions, and data are supported by quantitative and biochemical analysis, highlighting significant differences in the behavior of −SC and +SC molecules. The relevance of these results on the biological properties of DNA is discussed.

## Results and Discussion

### Effect of immobilization conditions on structural conformation of plasmid molecules with different supercoiling degree observed by AFM

**Figure 1:**
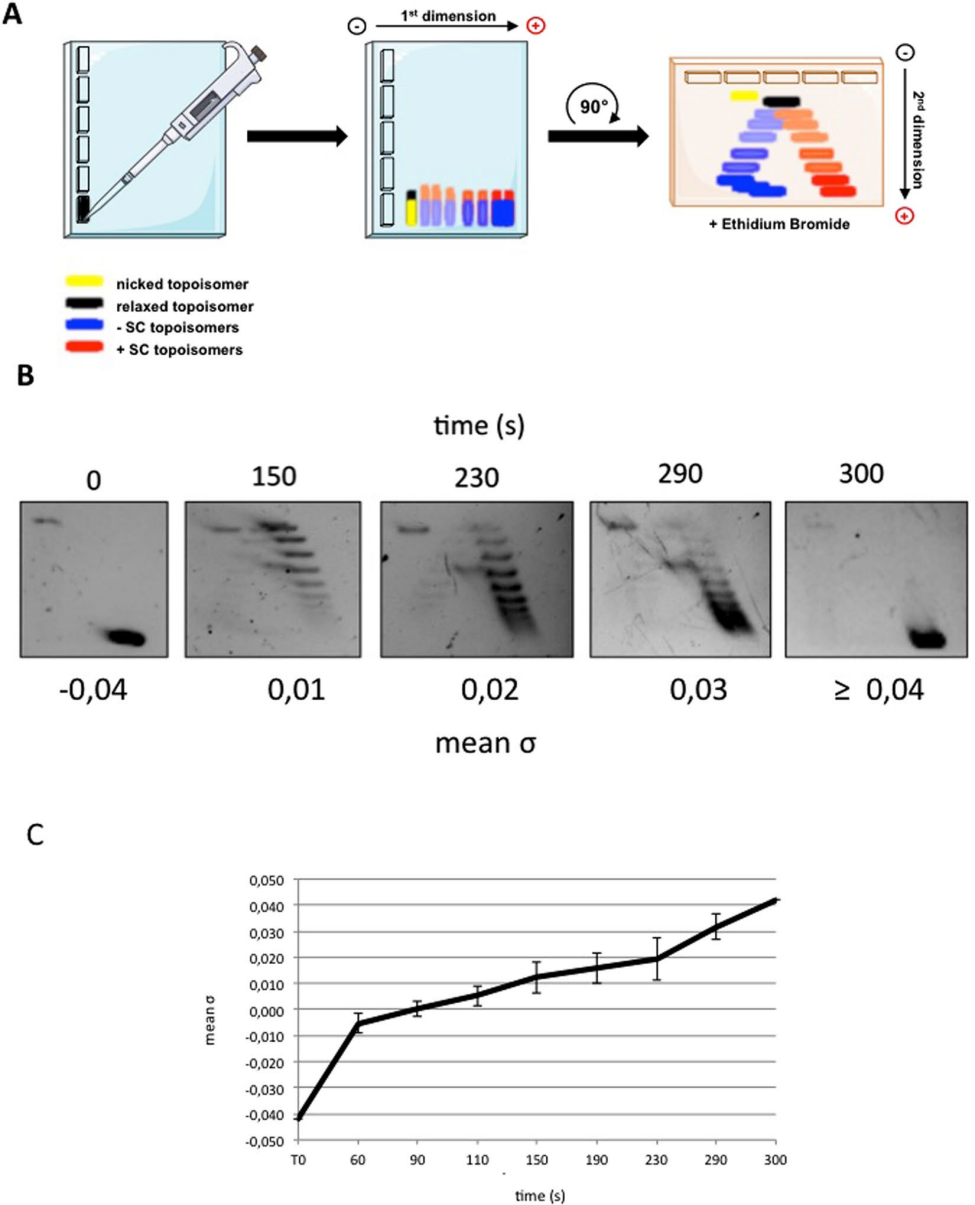
Reverse gyrase time course reaction analysed by 2D-gel electrophoresis. (**A**) Schematic representation of 2D-gel migration of −SC and +SC topoisomers. (**B**) −SC pBs DNA was incubated with *S*. *solfataricus* RG^30^ at 90 °C for the time indicated and subjected to 2D electrophoresis. For each time point, the mean σ was determined as described in Experimental Procedures. Representative gel is shown. (**C**) The graph shows increasing of positive supercoiling (expressed as mean σ) with time during RG reaction. The curve is not completely linear, due to the complexity of the reaction, which is affected by a multiplicity of parameters^30,31^. Data are from three independent experiments.

RG is a powerful tool to obtain plasmid molecules with different degree of supercoiling: indeed, by changing reaction parameters (temperature, time, DNA/enzyme ratio and so on) it is possible to modulate the enzyme activity and obtain distinct populations of topological isomers with defined ΔLk (called topoisomers; see Fig. 1A for a scheme of the reaction)^29,30^. Reactions were set up using the 3.000 bp pBlueScript plasmid (pBs), purified from *Escherichia coli* cultures in its negatively supercoiled form, and the RG from the archaeon *Sulfolobus solfataricus*^10–12,29^. In a time course experiment, aliquots of products were withdrawn after different incubation times and analysed by two-dimensional gel electrophoresis, enabling separation of −SC and +SC topoisomers (Fig. 1A). Figure 1B shows supercoiling modification of the pBs plasmid with time, from −SC (time 0) to +SC (300 sec.). The topological state of each topoisomer population was expressed calculating the mean specific linking number (mean σ) obtained from the σ values of the most abundant topoisomers produced in each reaction (see Methods). The mean σ of each topoisomer population was plotted as a function of time, showing the increase of plasmid supercoiling density during reverse gyrase reaction (Fig. 1C).

Among the time course products, four plasmid populations with defined mean σ (−0.04; +0.01; +0.03; ≥ +0.04) were selected, purified and analysed by AFM in air. Since the only images available so far for positive plasmids were obtained on silanized mica^28^, it is not known whether these molecules are affected by the deposition procedure, prompting us to explore different deposition methods. In order to increase the mica affinity for negatively charged molecules such as DNA, different strategies can be followed. Cation-assisted deposition is widely used due to the simplicity of sample preparation; it has been suggested that nucleic acids deposition on mica in the presence of divalent cations results in relatively weak binding and allows for the equilibration of molecules during deposition. A further drawback of this technique is that the cation requirement limits the range of experimental conditions that can be used. Alternatively, treatment of mica with aminosilanes is also an effective procedure for nucleic acids binding; silanized mica enables a stronger binding of nucleic acids and tends to trap DNA molecules in a configuration that corresponds to the 2D projection of their solution conformation^31,32^; however, hydrolysis of aminosilanes and aggregation is a complication associated with this technique.

We tested both procedures and explored a wide range of experimental conditions to find deposition protocols yielding reproducible binding of plasmids while preserving the specific features of molecules with different supercoiling degree. We selected appropriate conditions for deposition on mica either in the presence of MgCl_2_ or after treatment with 3-Amino propyl-trimethoxysilane (APTMS).

**Figure 2:**
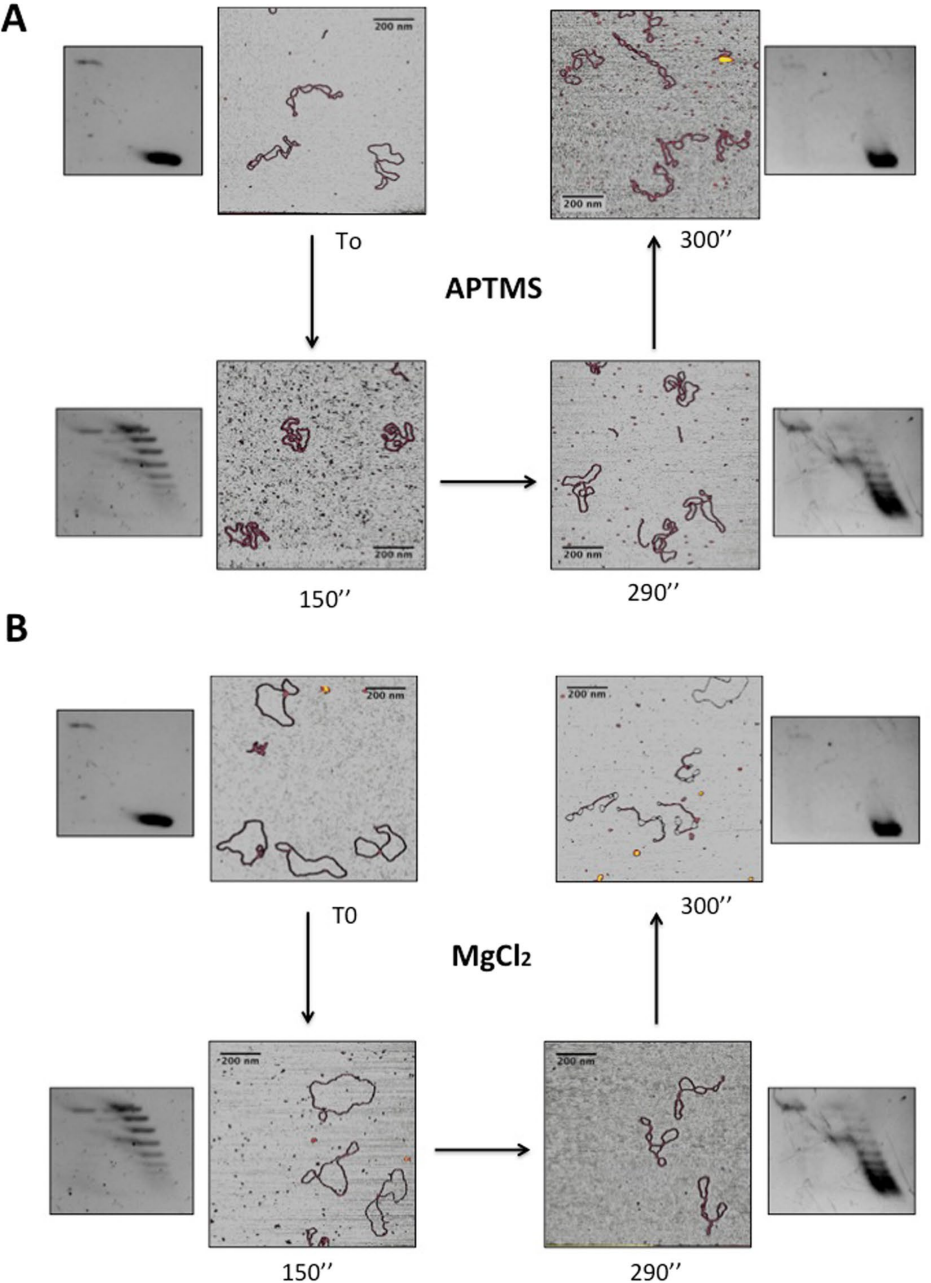
AFM imaging of distinct topoisomer populations deposited on mica under different conditions. (**A**) APTMS-treated mica or (**B**) in the presence of MgCl_2_. Aliquots of DNA were withdrawn during RG reaction at indicated reaction time and analyzed by AFM. Insets show the migration on 2D gel of each purified population at time 0 (σ = −0,04), after 150” (σ = +0,01), 290” (σ = +0,03) and 300” (σ ≥ +0,01) of reaction. The RG reaction products were monitored on 650 plasmids (about 65 plasmids for each timepoint).

When deposited on APTMS-treated mica in the presence of 100 mM NaCl, highly −SC plasmids (σ = −0.04) assumed a plectonemic form with several crossings seen in each molecule and, occasionally, irregularly balled-up or aggregated forms (Fig. 2A). These shapes were already reported for other plasmids having a similar supercoiling degree^33^. In contrast, the populations of topoisomers with an intermediate degree of supercoiling (+0.01 < mean σ < +0.03) lost the plectonemic form and showed a reduced number of crossings. Finally, when σ reached the value of +0.04, the molecules adopted again a highly plectonemic conformation (Fig. 2A).

Strikingly different results were obtained when the same samples were deposited in the presence of MgCl_2_. Highly −SC plasmids adopted a loose geometry, looking like open circular molecules or showing very few crossovers (Fig. 2B), similar to those reported by Lyubchencko and colleagues^34^. A clear transition from relaxed to plectonemic structures was observed at σ value of +0.04, as seen for the same molecules deposited on APTMS-treated mica. Thus, under these conditions the plectonemic aspect seems a peculiar feature of +SC molecules with a mean σ ≥ +0.04.

**Figure 3:**
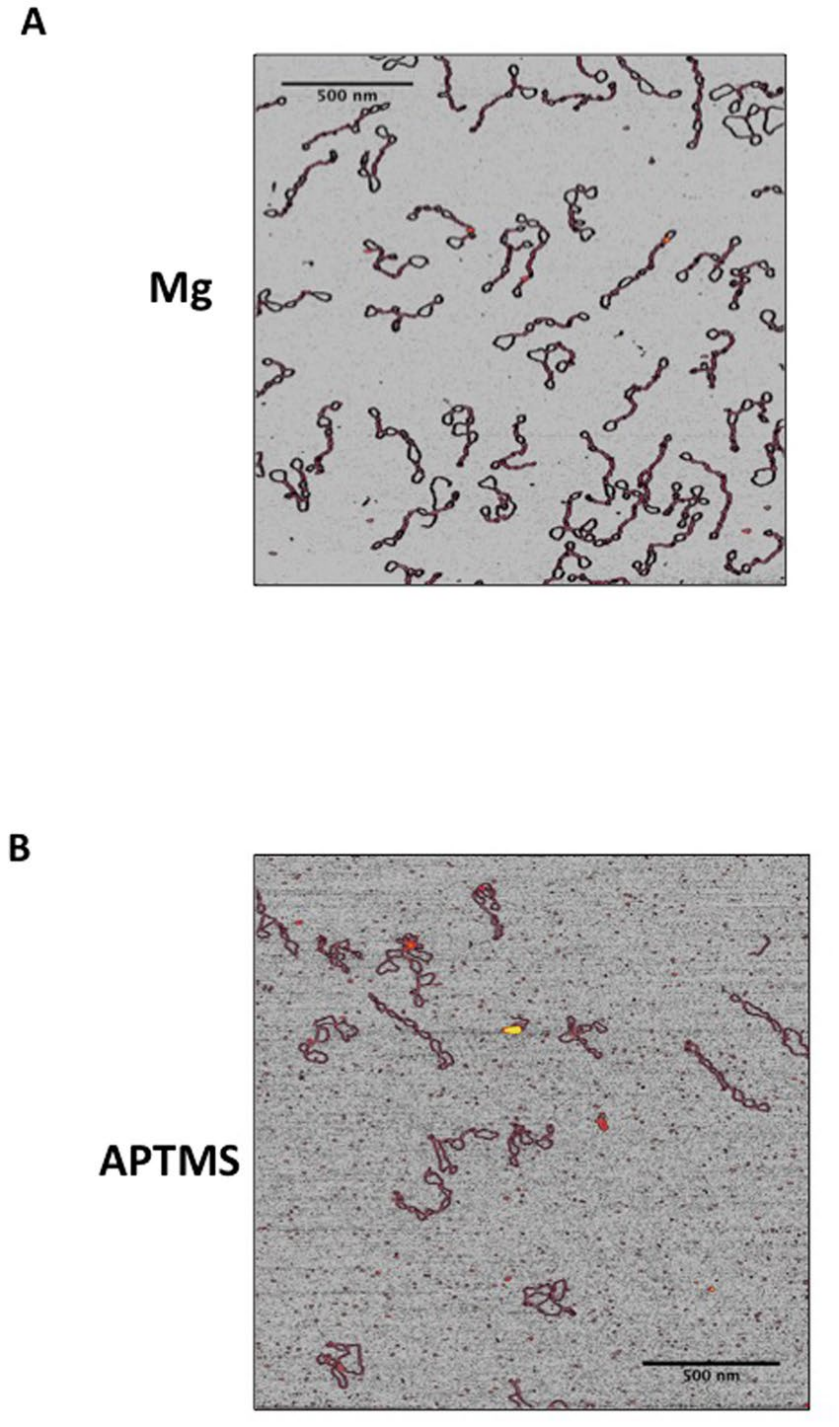
Comparison of the shapes of highly +SC plasmids (σ ≥ +0,04) deposited on mica under different conditions. (**A**) In the presence of 10 mM MgCl_2_; (**B**) APTMS-treated mica. Representative AFM images are shown.

Extensive comparison of the shapes of highly +SC molecules on the two surfaces showed that they appear highly homogeneous (Fig. 3), thus suggesting that the shape of these molecules is not heavily affected by the depositions conditions. Moreover, these images show that the end-point product of RG is a homogeneous population comprised of a single, or a few, topoisomer(s) with very close ΔLk.

To better understand the reasons of this striking difference, we acquired AFM images of linearized DNA plasmids on APTMS-treated-mica and on bare mica in the presence of MgCl_2_ (data not shown) and measured their contour length. The measured contour length of our DNA molecules on APTMS-treated-mica was 985 ± 40 nm, in very good agreement with the estimated length of this 3000 bp long DNA molecule in B form, which is 980.8 nm. In contrast, the experimental value obtained when DNA was deposited in the presence of Mg cations was of 893 ± 60 nm, with a decrease of about 9%. In a recently published paper, Lipiec *et al*.^32^ suggested that the local structure of DNA molecules deposited on aminosilane-treated mica resembles that of the B crystal form; in contrast, when molecules were deposited using the divalent cation adhesion method, a partial transition toward the A form occurs. If we take into account the structural parameters of the two DNA forms (A form: twist 32.7 degrees, rise 0.26 nm per base. B form: twist 34.3 degrees, rise 0.34 nm per base)^35^ it is possible to estimate the amount of B to A transition, by comparing the contour length of linear DNA in the two deposition conditions^31^. If we indicate with α the fraction of DNA molecules that switch from B to A form, we can compute the contour length by a linear combination of the two basic forms (see Equation 1),

1 $${L}={{N}}_{{bases}}\cdot {{R}}_{{B}}(1-{\alpha })+{{N}}_{{bases}}\cdot {{R}}_{{A}}\cdot {\alpha }$$

where *N*_*bases*_ is the number of bases per molecule, and *R*_*B*_ and R_A_ are the helical rise per base in the B and A forms, respectively. Inverting this equation, we can calculate α from the contour length of DNA deposited on APTMS-treated mica (which is considered to be in the B form), and the length of DNA deposited in presence of MgCl_2_. From our results we obtain a value α = 0.4, in good agreement with the published value of 0.39^32^.

As noted above, A and B forms also differ in the helical pitch. In closed circular DNAs, a transition between these two forms affects the balance between twist (Tw) and writhe (Wr) to maintain a constant Lk^36,37^.

Although the great complexity of this system prevented us from quantitatively describe the different plasmids shapes, our experimental results support the idea that the B to A transition might be one reason for the different topologies they assume when deposited using the two methods. In a relaxed plasmid $L{k}_{0}=T{w}_{0}$, where $T{w}_{0}={N}_{bases}/(bases/turn)\,\,$is the “natural” twist and it equal to $T{w}_{0}^{B}=286$ for pure B-DNA and to $T{w}_{0}^{h}=281$ for hybrid form. We compared these values with the $Lk$ of the two extreme states of our supercoiled molecules, i.e. pBlueScript plasmids in their negatively supercoiled form (−SC_0_) and after 300 seconds of RG reaction (+SC_300_), which were: $Lk=274$ and $Lk=297$, respectively. We could then estimate how much supercoiling needs to be accommodated by each type of plasmid if deposited on either surface; the results are summarized in Table 1.

**Table 1:** linking number differences *ΔLk* between relaxed plasmid states (*Lk*_0_) and the plasmids in closed form (*Lk*), for the two supercoiling states and for the two deposition conditions.

${\rm{\Delta }}Lk=Lk-L{k}_{0}$	−SC_0_	+SC_300_
APTMS (pure B-DNA)	274–286 = −12	297–286 = +11
MgCl_2_ (hybrid form)	274–281 = −7	297–281 = +16

The data indicate that deposition of supercoiled plasmids in the presence of Mg ions leads to a transition toward the DNA A form, which is responsible for the a smaller *ΔLk* as it decreases by 5 absolute units compared to the APTMS case (−7 vs −12). As a consequence, SC- plasmids resulted less negatively supercoiled, whereas SC+ were more positively supercoliled, as observed in Figs 2A,B and 3.

In order to give statistical significance to our data, we sought to perform quantitative analysis of the structural conformations adopted by the four plasmid populations. As pointed out by Irobalieva *et al*.^27^, 386 bp long plasmids arrange into a number of different configurations. Because the energy required to supercoil a plasmid is inversely related to its length, longer molecules show even broader range of configurations. Thus, a detailed analysis of the structural conformations may require algorithms currently not available. We developed a numerical routine that traces plasmids from AFM images and extracts a set of geometrical parameters able to discriminate the sign of the supercoiling. While common geometrical descriptors (asphericity, eccentricity, solidity, etc.) were not effective in classifying supercoiling sign (data not shown), we found that the following two parameters quantitatively described the different topoisomer populations:

- density: defined as the ratio between the area occupied by the plasmid and the entire area of the convex hull defining the plasmid;
- Euler number: defined as the number of objects minus the numbers of holes they contain (essentially one object, the plasmid, minus the number of holes contained within it).

**Figure 4:**
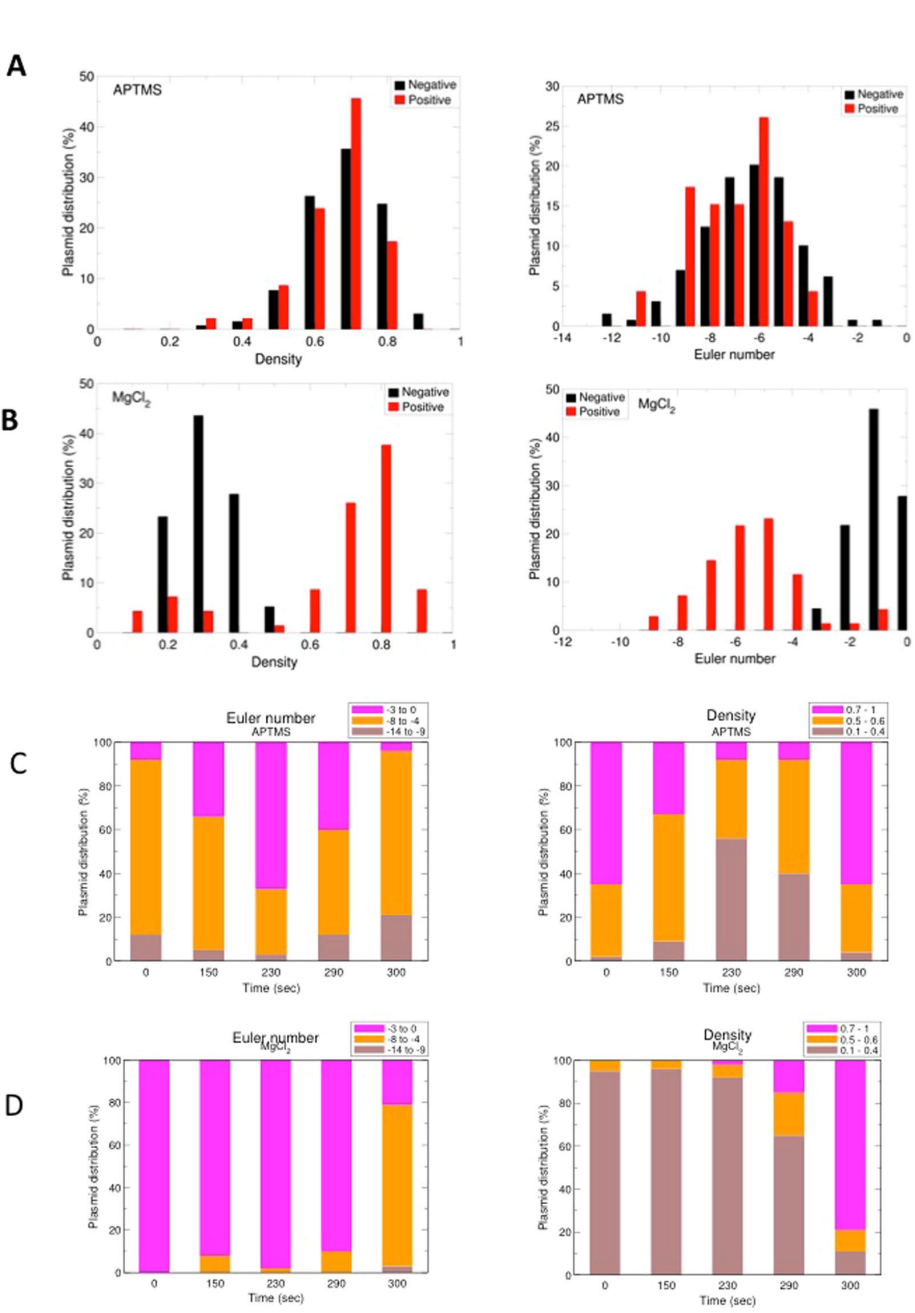
Quantitative analysis of supercoiled molecules. Density and Euler number were extracted from AFM images. (**A**,**B**) Density and Euler number of highly −SC and +SC molecules on APTMS-treated surfaces (**A**) and in the presence of Mg^2+^ ions (**B**). (**C**,**D**) Density and Euler number values for all five plasmid populations obtained during RG reaction, on APTMS-treated surfaces (**C**) and in the presence of Mg^2+^ ions (**D**). On APTMS, supercoiled molecules show higher densities, while more relaxed plasmids assume less dense shape. Similarly, the Euler number assumes the largest modulus for the most supercoiled forms, while the minimum modulus is obtained for relaxed plasmids. In contrast, deposition using MgCl_2_ produces a monotonic trend in both density and Euler number the −SC family (at 0 s) has a Euler number of −1 and low densities, while both Euler number modulus and density increase with increasing RG reaction time.

We found that higher density and lower Euler number were associated with increased plectonemic character: highly +SC and −SC populations deposited on APTMS-treated mica showed overlapping density and Euler number, consistent with their supercoiled aspect (Fig. 4A); in contrast, both parameters were able to discriminate between the two populations observed in the presence of MgCl_2_ (Fig. 4B). This fact suggests that −SC and +SC plasmids assume different shapes because of specific interactions with the Mg^2+^ cations. Plasmid populations with intermediate ΔLk showed intermediate plectonemic character when deposited on APTMS-treated mica (Fig. 4C), whereas all populations except the highly +SC looked essentially relaxed on MgCl_2_ (Fig. 4D).

Taken together, these results suggest that, whereas the possibly 3D- shape of highly −SC plasmids is dependent on the deposition conditions, the structural configuration of +SC molecules is relatively insensitive to deposition conditions; in particular, highly +SC plasmids maintain their plectonemic arrangement under conditions that determine transition of −SC and intermediate topoisomers to looser geometry.

These results are the first experimental demonstration of the braiding effect induced by freely diffusing divalent cations, and support a potential role of direct DNA–DNA interactions in tuning chromatin compaction^38,39^. In fact, previous results obtained by molecular dynamics simulation demonstrated that the opposite chirality of superhelices (positive for −SC and negative for +SC) produces different types of inter-segmental interactions along the plectonemes: +SC induces right-handed crossovers characterized by a strong groove-backbone interaction (i.e. the major groove of one helix interlocks with the minor groove of the other), while −SC supercoiling produces a simple juxtaposition of the grooves (i.e. major-major groove overlap)^40^.

It is known that length is an important factor affecting DNA morphological and physical parameters. For instance, AFM analysis of long (>15 Kbp up to 45 Kbp) −SC molecules showed that these molecules adopt compact, highly ordered interwound filaments, called hyperplectonemes^41^. Although no data on +SC molecules of comparable size are available, it is likely that length might affect the structure of these molecules as well, and further investigation on this point is warranted.

### AFM Imaging in liquid of −SC and +SC DNA molecules

**Figure 5:**
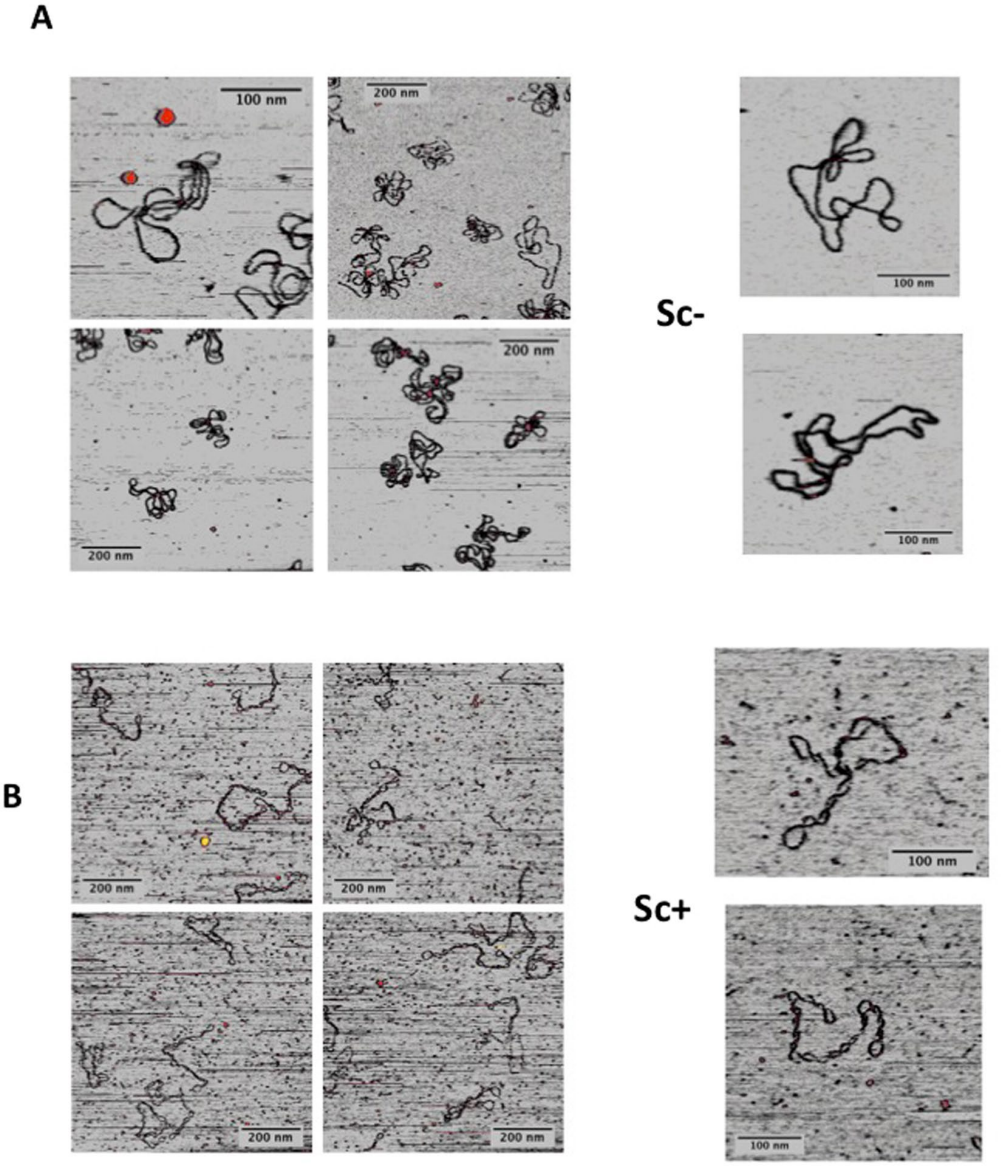
AFM analysis of supercoiled plasmids in solution. Representative images of −SC (**A**) and +SC (**B**) molecules at 35 °C are shown.

Our AFM images in air showed that only −SC molecules are heavily affected by the deposition procedure, while +SC shapes are definitely more stable. Since DNA may change its conformation during the steps of sample preparation and deposition, observation of molecules in solution may better reflect their conformation under physiological conditions and help overcome possible structure alterations arising from the rinsing and drying of the sample. Moreover, observation in liquid can be carried out under a number of controlled conditions. No data on +SC DNA in solution are available so far. We thus set up the experimental conditions for direct observation of −SC (σ = −0.04) and +SC (σ ≥0.04) DNA molecules in aqueous solutions in the presence of mild ionic strength (100 mM NaCl). Under these conditions, the shapes of both −SC and +SC molecules looked very similar to those observed on APTMS in air (Fig. 5A and B).

Assuming that both deposition methods permit molecular equilibration, the experimental data show that −SC, but not +SC plasmids were affected by the deposition conditions. The wounded (but not plectonemic) shapes assumed by −SC can be related to a greater flexibility of the −SC with respect to the tight arrangement assumed by the +SC. These forms resemble the hyperplectonemes recently described^41^ and can be the result of a negligible braiding effect: the distribution between Wr and Tw are mainly due to the mechanical stress induced by supercoiling. On the other hand, +SC molecules exploit the synergistic effect of both steric and electrostatic interactions that favour braiding and development of plectonemes. This is a quite unexpected and novel result, since it demonstrates a clear asymmetric behaviour of plasmids with different SC sign.

### Temperature effect on plasmids supercoiling

It is well known that temperature is an important parameter affecting DNA topology. However, the effect of temperature on supercoiling has been analysed by AFM only for −SC plasmids in air^42^; this study showed that increasing temperature from 25 to 50 °C induces a shift of the plasmid structure from supercoiled to more relaxed shapes. Moreover, observation at higher temperatures was accompanied by the appearance of globular structures, which became predominant at 80 °C^43^. These structures were interpreted as a result of denaturation of localized regions, followed by collapse of the single strands. Finally, at 100 °C DNA was degraded into small fragments^43^. Since these experiments were performed in air, it is not known whether these structures might be due to deposition conditions. Moreover, no data on the effect of high temperature on +SC DNA are available.

**Figure 6:**
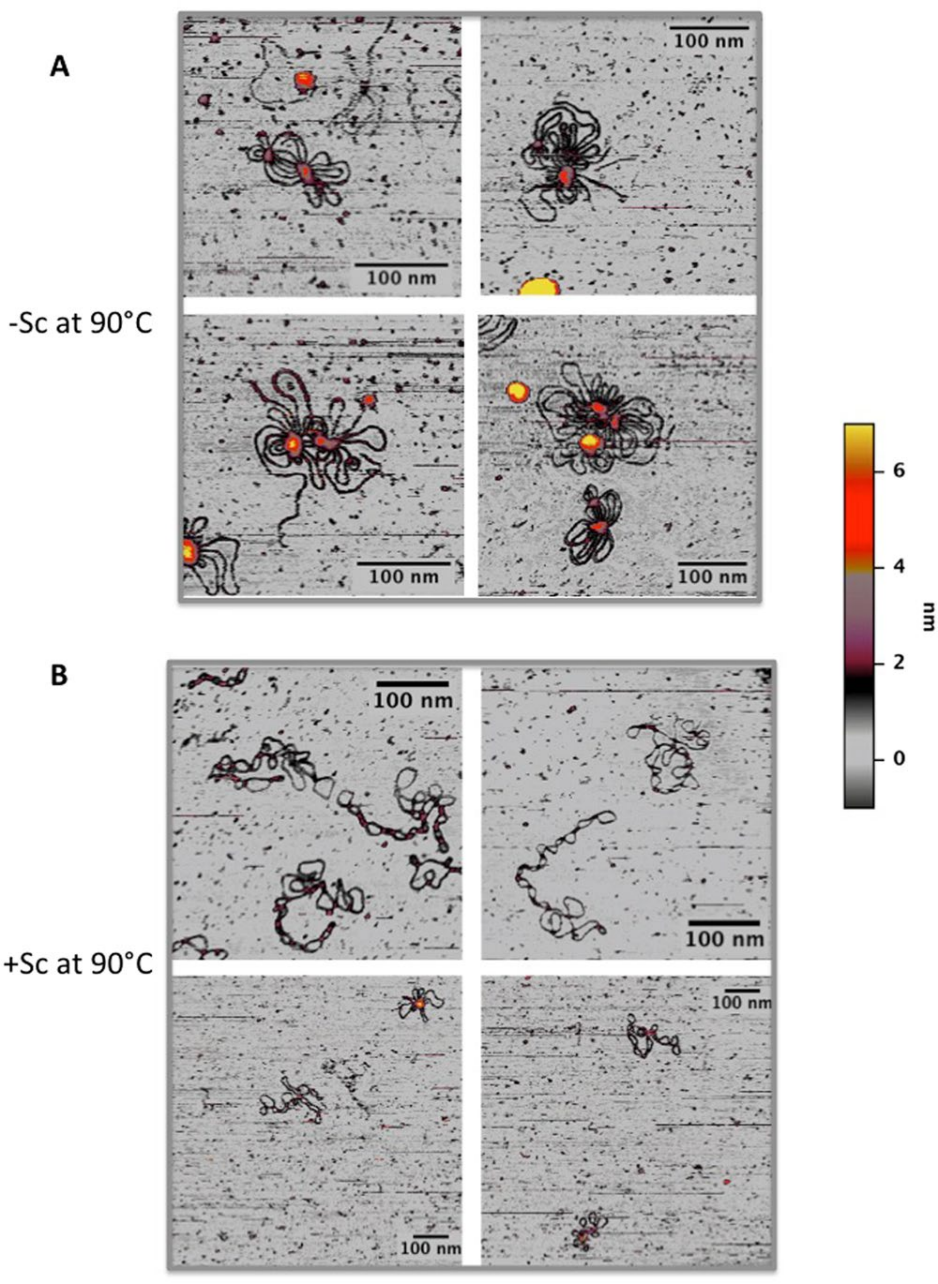
AFM analysis of supercoiled plasmids in solution at high temperature. (**A**) −SC +  in liquid at 90 °C. Plasmids lost their plectoneme shape and assume a globular form compatible with denaturation of their structure. (**B**) +SC plasmid in liquid at 90 °C. Nearly all plasmids maintain their plectonemic shape, thus confirming a more stable behavior of these molecules against thermal denaturation.

We thus sought to set up a protocol where plasmid solutions were pre-incubated at a given temperature, deposited on mica-APTMS and quickly observed in solution at the same temperature. Since the APTMS layer might be partially hydrolysed at high incubation temperatures^44^ reducing the binding of plasmids to the mica surface, the incubation time of the substrate at high temperature was reduced to a minimum. As shown in Fig. 6, after pre-incubation for 10 min at 90 °C and direct observation after additional 3 minutes on mica-APTMS, both −SC and +SC plasmids were still bound to the treated mica surfaces, suggesting a reasonable stability of the APTMS layer in our conditions. Interestingly, under these conditions SC molecules assumed globular and aggregated structures, confirming previous observations in dried samples^43^ (Fig. 6A). In striking contrast, +SC molecules conserved their plectonemic structure almost intact after the same treatment (Fig. 6B; for comparison purposes AFM images of the same pool of plasmids acquired at 35 °C are reported in Fig. S1). Again, this behavior confirms that +SC plasmids topology can withstand external changes, while the −SC behavior is compatible with a more flexible polymer.

**Figure 7:**
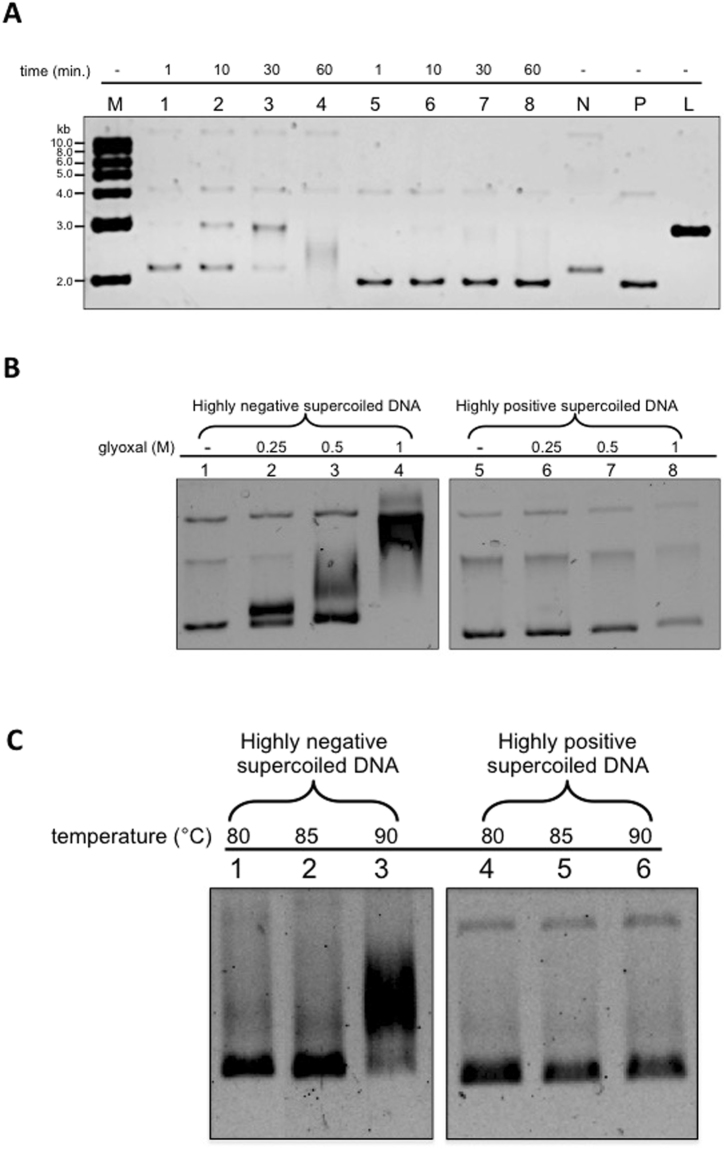
Biochemical analysis of supercoiled plasmids. (**A**) Plasmid DNA preparations were incubated with nuclease Bal-31 for increasing timespans, as indicated lanes 1–4, −SC plasmid; lanes 5–8, +SC plasmid. N mock-incubated −SC DNA; P mock-incubated highly +SC DNA, L linearized 3000 bp plasmid; M 1 kb DNA ladder. Over time, samples were removed, quenched by the addition of stop buffer and the products analysed on 1% agarose gel. (**B**) Incubation of plasmid DNA with glyoxal. Highly −SC and +Sc DNA preparations were incubated overnight in the absence or presence of increasing concentrations of glyoxal, as indicated, and analyzed on 0.8% agarose gel. The lanes 1–4 (−SC plasmid) and 1–8 (+SC plasmid) are cropped from different parts of the same gel. (**C**) Effect of temperature on glyoxal binding. Highly −SC and +SC DNA were incubated for 5 min. at indicated temperatures in the presence of 1 M glyoxal and analyzed by 0.8% agarose gel electrophoresis. The lanes 1–3 and 4–5 are cropped from different parts of the same gel.

Previous experiments showed that, whereas −SC molecules (minicircles as well as plasmids) expose single strand regions even at room temperature, +SC molecules do not^27,28^. We set up a similar experiment with our molecule populations: when incubated with the ssDNA endo-exonuclease Bal31 (which is able to cut single strand DNA in denatured bubbles), −SC DNA was degraded completely after 1 hour at 30 °C (Fig. 7A, lane 1–4), whereas highly +SC plasmids (σ ≥ +0,04) were resistant to Bal31 digestion, confirming that they do not expose single strand regions at this temperature (Fig. 7A, lane 5–8).

Since our AFM analysis showed a striking structural stability of +SC molecules even at 90 °C, we sought to find an alternative biochemical tool to probe the 2D structure of our molecules at high temperature. To this aim we used glyoxal, a small molecule that traps exposed bases and is resistant to high temperature. When incubated with 1 M glyoxal at room temperature, the −SC DNA band was shifted, indicating binding of the drug to single strand regions (Fig. 7B, lane 1–4); in contrast, the mobility of the +SC DNA was not affected when incubated with increasing concentrations of glyoxal (Fig. 7B, lane 5–8). In order to evaluate the effect of temperature, −SC and +SC DNA molecules were incubated for 5 minutes at increasing temperatures (from 80 °C up to 90 °C) in the presence of glyoxal. As expected, for the underwound DNA the glyoxal-induced shift increased with temperature (Fig. 7C, lane 1–3), because temperature increase further induces local denaturation. Interestingly, +SC plasmids did not bind glyoxal at any temperature up to 90 °C (Fig. 7C, lane 4–6) indicating that they do not contain unpaired bases when incubated for 5 min up to 90 °C and are highly resistant to thermal denaturation.

In a similar analysis performed on DNA minicircles, Irobalieva and colleagues found that topoisomers with ΔLk ranging from −6 to −1 showed exposed bases, whereas those with ΔLk = 0, +1 and +2 did not, in line with our results. However, they also found that the +3 topoisomer was sensitive to Bal31 degradation, albeit at lesser extent as compared with −SC DNA molecules. This result was explained assuming that overwinding induces a sharp bending in this small topoisomer, resulting in local denaturation^27^. In contrast, we could not observe any evidence of denaturation in our +SC with very high ΔLk (≥ +12), suggesting that these larger molecules can accommodate the Wr excess without the need of bending or denaturation to compensate for the torsional stress.

## Conclusion

**Figure 8:**
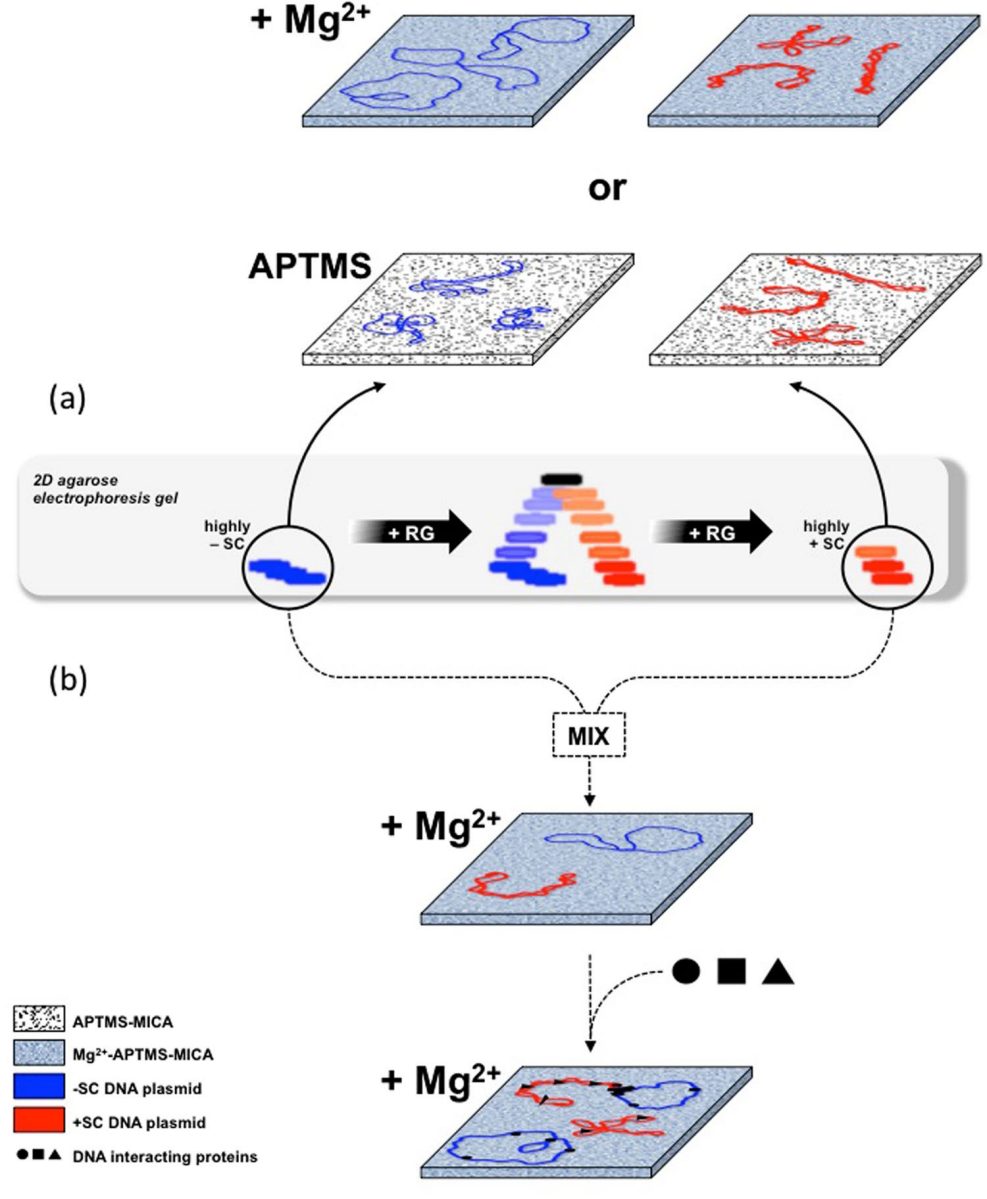
Schematic representation of −SC and +SC plasmids analysed by AFM results obtained in this study **(a**) and their potential application to study DNA topology and its interactions with other molecules upon MgCl_2_ deposition (**b**).

In this work we exploited AFM to perform for the first time a systematic analysis of plasmids having excess linking number between −12 to ≥ +12 (|σ|≈ 0.04), covering the full range of physiologically relevant topological states. By exploiting the high resolution imaging of DNA we studied how plasmid conformation is affected by immobilization, surface and temperature. We found a strong asymmetry in the behaviour of −SC and +SC molecules (summarized in Fig. 8a). Both −SC and +SC molecules deposited and dried onto APTMS-treated surfaces, assumed plectonemic shapes. In contrast, the presence of cations (MgCl_2_) during deposition influenced the shape of −SC molecules, which assumed an open conformation, but not of +SC molecules, possibly due to a specific interaction of DNA with Mg cations on surfaces which can alter the canonical B structure or maybe suggesting that positive overwinding protects DNA from cations-dependent alterations. Moreover, direct observations in liquid showed a remarkable structural stability of +SC molecules at high temperature, whereas −SC molecules collapsed above 90 °C. These data, confirmed by biochemical analysis, are in line with the biological role of both negative supercoiling, which is thought to facilitate breathing of the double helix, and positive supercoiling, which is likely to contribute to DNA stability under extreme conditions.

Showing that the opposite signs of DNA affect the physical properties of the superhelices, our study provides new insights that may contribute to understand the role of specific DNA topology in the cellular environment as well as the physiological function of reverse gyrase and its hallmark reaction^45,46^. Our results also show that AFM is a powerful tool to correlate different shapes of DNA molecules to different topologies, upon deposition in the presence of MgCl_2_. For instance, by AFM it is possible to analyze, identify and quantify at single molecule level: (i) DNA samples with mixed topologies; (ii) different local topologies in DNA-protein complexes: (iii) supercoiling-dependent binding of specific proteins (Fig. 8b). Our results might also be useful to explore DNA dynamics and its interactions in confined space^47–49^ with other charged molecules, such as chemotherapeutic compounds, intercalating agents, and proteins involved in DNA metabolism.

In particular, the superhelical density of DNA is a critical determinant of the nature of bound proteins and the assembly of nucleoprotein complexes. Topology-dependent binding enables DNA-binding proteins to discriminate between different sites in DNA^50^. For instance, HMG–containing and TATA binding proteins bind to untwisted DNA, and bacterial architectural proteins, such as HU and H-NS, as well as eukaryotic histones, bind and stabilize -SC DNA^51,52^. Yet others, for example the bacterial FIS protein preferentially binds intermediate supercoiled DNA as compared with the extremes of the topological spectrum^53^; the reverse is true for the tumor suppressor p53, which binds preferentially both −SC and +SC DNA, whereas it shows lower affinity for relaxed DNA^54^. Finally, it will be interesting to investigate the interplay between DNA knots, which are occasionally introduced by topoisomerases, and +SC. The behavior of knots is an important avenue of future research, because they heavily affect nucleic acids properties both *in vivo* (e.g., transcription and replication), and in single-molecule studies, such as pore translocation^55^.

## Methods

### RG time course reaction

For time course experiments, we incubated the RG from *S*. *solfataricus*^30^ (80 nM) with 3000 bp long −SC pBs plasmid (10 nM) in a final volume of 150 μL of 1 ×  RG buffer (35 mM Tris-HCl, pH 7.0, 0.1 mM Na_2_EDTA, 30 mM MgCl_2_, 2.0 mM DTT, 1 mM ATP). Reactions were incubated at 90 °C and withdrawals of 30 μL were done at different times as indicated. After incubation, DNA samples were purified by phenol extraction and ethanol precipitation; an aliquot (300 ng) of each sample was analysed by two-dimensional gel electrophoresis in 1.2% agarose and 1×  Tris borate-EDTA buffer^41^. Gels were stained with ethidium bromide (1 μg ml^−1^) and destained in deionized water for the analyses with UV light by a VersaDoc 4000™ apparatus and the QuantityOne software (Bio-Rad). Another aliquot of the same samples was used for AFM analyses.

### Determining supercoiling density of plasmid DNA molecules

Topological properties of DNA are defined by: twist (Tw, the number of times each helix twists around the other) and writhe (Wr, the number of crossings the double helix makes around itself); in a covalently closed DNA molecule, the sum of these two parameters is a topological invariant, called linking number (Lk = Tw + Wr). Because the energy required to supercoil a polymer depends on its length, a useful parameter to compare different topological isomers (topoisomers) is the supecoiling density σ = (Lk − Lk_0_)/Lk_0_, where *Lk*_0_ and *Lk* represent the DNA linking number for the relaxed and the supercoiled DNA, respectively. The superhelical density of topoisomers with different supercoiling degree was estimated by 2D gel electrophoresis. Briefly, after migration the gel was stained with ethidium bromide (1 μg ml^−1^), destained, and photographed under UV light. The relative intensity of each band was measured and the DNA linking number change (ΔLk) of corresponding topoisomers was determined and used to obtain σ values applying the above equation. The σ values of all topoisomers whose intensity was >30% of the intensity of the most abundant topoisomer were used for the calculation of the mean σ value.

To increase the resolution of highly negatively supercoiled molecules, which run as single bands in 2D gels, samples was subjected to monodimensional electrophoresis in the presence of a small concentration of the intercalating agent chloroquine, which causes a decrease in Tw and consequently an increase of Wr. Under these conditions, the band of the negative plasmid was split into a few bands, with ΔLk ranging from −7 to −12. The σ of the most −SC topoisomer (ΔLk = −12) is −0,04, in line with the supercoiling density of plasmids extracted from *E*. *coli* cells cultures^42^ (Fig. S2).

For the +SC topoisomer population, monodimensional electrophoresis without intercalants was used, showing that they still migrated as a single band, whose σ was ≥ 0,04 and it was not possible to determine whether this population is comprised of a single or a few compact topoisomers.

### DNA deposition protocol

For all samples, DNA stock solution was mixed with ultrapure water, Tris buffer at pH 7.5 (final concentration 7 mM) and salts (see below), achieving a final DNA concentration of 2 ng μL^−1^. To induce a reproducible and attractive interaction between the plasmids and the surface of the mica we either added MgCl_2_ (3 mM final concentration) to the solution or we added an APTMS layer to mica before DNA incubation. APTMS- treated mica was obtained exposing freshly cleaved mica substrates to APTMS vapors in vacuum for 3 minutes and used immediately. In this latter case NaCl at a final concentration of 100 mM was added to the mixture. For measurements in air, 20 μL of the final plasmid solution were added to the mica and incubated for 3 min, afterward samples were gently rinsed with 0.6 ml of ultrapure water and dried under a mild air flow.

For measurements in liquid, two different procedures were used, both exploiting APTMS-treated-mica as substrate. For measurements at low temperature (35 °C), NaCl was added to the mixture at a final concentration of 100 mM, and 20 μL of the final plasmid solution were incubated with mica for 3 minutes. Then, samples were gently rinsed adding and removing 80 μL of the buffer solution used to dissolve the plasmids. For high temperature measurements, plasmid solutions were heated at 90 °C for 10 minutes, then 25 μL of this solution were incubated with the APTMS-treated mica substrate at 90 °C for 3 minutes, supplying additional buffer to compensate for evaporation and avoid concentration effects. The short incubation time was chosen to reduce hydrolysis of aminosilane, which may occur at high temperature, although at slow rate^44^. Finally, the incubated mica was gently rinsed (at room temperature) adding and removing 80 μL of the same buffer solution used to dissolve the plasmids. No salt was added in this case to increase plasmid affinity toward mica at this high incubation temperature. In both cases, 100 μL of the corresponding buffer were added to the sample before insertion in the AFM cell.

### AFM Imaging

AFM images were collected using an Asylum Research Cypher equipped with an environmental scanner. Details of imaging parameters are the following:

- Imaging in air: NSG 01 DLC (nominal parameters: force constant 5.1 N/m, resonant frequency 150 kHz) and Olympus 240 TS (nominal parameters: force constant 2 N/m, resonant frequency 70 kHz) tips. No main differences were found using these two tip models. AFM fast scan rate was around 1–1.5 Hz with a tip oscillation amplitude below 10 nm.
- Imaging in liquid: Olympus BL-AC40TS (nominal parameters: force constant 0.09 N/m, resonant frequency 110 kHz in air) tip. Fast scanning rate between 2 and 4 Hz, tip oscillation amplitude between 2 and 4 nm.

Images were obtained at several separate mica locations. All the AFM acquisitions in air were performed at room temperature, while acquisitions in liquid were performed at 35 °C. Asylum provided software was used for data acquisition. AFM visualization was performed using ImageJ^26^ and dedicated plug-ins that we developed.

### Nuclease Bal-31 assay

−SC and +SC plasmid DNA (400 ng in 40 μL final volume) was incubated with 0.2 units of nuclease Bal-31 at 30 °C in 20 mM Tris-HCl, pH 8.0, 600 mM NaCl, 12 mM MgCl_2_, 12 mM CaCl_2_ and 1 mM disodium EDTA. At 1, 10, 30 and 60-minute intervals, 10 μL (100 ng) samples were removed, stopped by addition of an equal volume of stop buffer (50 mM Tris-HCl, pH 8.0, 100 mM disodium EDTA, 10% glycerol, 200 μg ml^−1^ proteinase K), followed by incubation at 45 °C for 30 min to degrade Bal-31. Products were analyzed by electrophoresis through 1.2% agarose in 1× Tris-Borate EDTA. Gels were stained with ethidium bromide (1 μg ml^−1^) and destained in deionized water for the analyses with UV light by a Bio-Rad VersaDoc apparatus.

### Glyoxal assay

Glyoxal was first deionized with AG-501-X8 mixed bed ion-exchange resin (Bio-Rad, Hercules, CA). −SC and +SC plasmid DNA (100 ng) was incubated with increasing concentrations of glyoxal (from 0.25 to 1 M) in 10 mM sodium phosphate, pH 7.0, for 16 h at room temperature. Control reactions were incubated in 10 mM sodium phosphate only. Samples were analysed by electrophoresis through 0.8% agarose gel in 1× Tris-Borate EDTA. Gel was analysed as already reported.

In order to determine the effect of temperature on glyoxal binding, we performed the reaction by incubating 100 ng of negatively or positively supercoiled DNA in presence of 1 M glyoxal for 5 min. at indicated temperatures. After incubation, samples were analysed as described above.

## Electronic supplementary material

Supplementary Figures and legends

## References

[1] Champoux JJ (2001). DNA topoisomerases: structure, function, and mechanism. Annu Rev Biochem..

[2] Chen SH, Chan NL, Hsie TS (2013). New mechanistic and functional insights into DNA topoisomerases. Annu Rev Biochem..

[3] Forterre P, Gribaldo S, Gadelle D, Serre MC (2007). Origin and evolution of DNA topoisomerase. Biochimie..

[4] Schoeffler AJ, Berger JM (2008). DNA topoisomerases: harnessing and constraining energy to govern chromosome topology. Q. Rev. Biophys..

[5] Nitiss JL (2009). DNA topoisomerase II and its growing repertoire of biological functions. Nat. Rev. Cancer..

[6] Vos SM, Tretter EM, Schmidt BH, Berger JM (2011). All tangled up: how cells direct, manage and exploit topoisomerase function. Nat Rev Mol Cell Biol..

[7] Wang JC (2002). Cellular roles of DNA topoisomerases: a molecular perspective. Nat Rev Mol Cell Biol..

[8] Koster DA, Crut A, Shuman S, Bjornsti MA, Dekker NH (2010). Cellular strategies for regulating DNA supercoiling: A single-molecule perspective. Cell..

[9] Seol Y, Neuman KC (2016). The dynamic interplay between DNA topoisomerases and DNA topology. Biophys Rev..

[10] Perugino G, Valenti A, D’amaro A, Rossi M, Ciaramella M (2009). Reverse gyrase and genome stability in hyperthermophilic organisms. Biochem. Soc. Trans..

[11] Vettone A, Perugino G, Rossi M, Valenti A, Ciaramella M (2014). Genome stability: recent insights in the topoisomerase reverse gyrase and thermophilic DNA alkyltransferase. Extremophiles..

[12] Visone V (2014). Chromatin structure and dynamics in hot environments: architectural proteins and DNA topoisomerases of thermophilic archaea. Int. J. Mol. Sci..

[13] Lulchev P, Klostermeier D (2014). Reverse gyrase: recent advances and current mechanistic understanding of positive DNA supercoiling. Nucleic Acids Res..

[14] Lipscomb GL, Hahn EM, Crowley AT, Adams MWW (2017). Reverse gyrase is essential for microbial growth at 95 °C. Extremophiles.

[15] Valenti A, Perugino G, Rossi M, Ciaramella M (2011). Positive supercoiling in thermophiles and mesophiles: of the good and evil. Biochem Soc Trans..

[16] Gilbert N, Allan J (2014). Supercoiling in DNA and chromatin. Curr. Opin. Genet. Dev..

[17] Postow L (2001). Positive torsional strain causes the formation of a four-way junction at replication forks. J. Biol. Chem..

[18] Bankhead T, Kobryn K, Chaconas G (2006). Unexpected twist: harnessing the energy in positive supercoils to control telomere resolution. Mol. Microbiol..

[19] Furuyama T, Henikoff S (2009). Centromeric nucleosomes induce positive DNA supercoils. Cell..

[20] Hirano T (2012). Condensins: universal organizers of chromosomes with diverse functions. Genes Dev..

[21] Meng H, Bosman J, Heijden T, Noort J (2014). Coexistence of twisted, plectonemic, and melted DNA in small topological domains. Biophys. J..

[22] Strick TR, Allemand JF, Bensimon D, Croquette V (1996). The elasticity of a single supercoiled DNA molecule. Science.

[23] Nagami F, Zuccheri G, Samorì B, Kuroda R (2002). Time-lapse imaging of conformational changes in supercoiled DNA by scanning force microscopy. Anal Biochem..

[24] Lyubchenko YL (2011). Preparation of DNA and nucleoprotein samples for AFM imaging. Micron..

[25] Ayache, J., Beaunier, L., Boumendil, J., Ehret, G. & Laub D. Artifacts in Transmission Electron Microscopy *in Sample Preparation Handbook for Transmission Electron Microscopy*, Springer (2010).

[26] Schneider CA, Rasband WS, Eliceiri KW (2012). NIH Image to ImageJ: 25 years of image analysis. Nature Met..

[27] Irobalieva RN (2015). Structural diversity of supercoiled DNA. Nat. Commun..

[28] Li D, Lv B, Wang Q, Liu Y, Zhuge Q (2017). Direct observation of positive supercoils introduced by reverse gyrase through atomic force microscopy. Bioorg Med Chem Lett..

[29] Valenti A (2008). Dissection of reverse gyrase activities: insight into the evolution of a thermostable molecular machine. Nucleic Acids Res..

[30] Bizard A, Garnier F, Nadal M (2011). TopR2, the second reverse gyrase of Sulfolobus solfataricus, exhibits unusual properties. J. Mol. Biol..

[31] Japaridze A (2016). Toward an Effective Control of DNA’s Submolecular Conformation on a Surface. Macromolecules..

[32] Lipiec E, Japaridze A, Szczerbinski J, Dietler G, Zenobi R (2016). Preparation of Well-Defined DNA Samples for Reproducible Nanospectroscopic Measurements. Small.

[33] Nagami F, Zuccheri G, Samorì B, Kuroda R (2002). Time-Lapse Imaging of Conformational Changes in Supercoiled DNA by Scanning Force Microscopy. Analytical Biochemistry.

[34] Lyubchenko YL, Shlyakhtenko LS (1997). Visualization of supercoiled DNA with atomic force microscopy *in situ*. Proc. Natl. Acad. Sci..

[35] Potaman, V. N. & Sinden R. R. DNA: Alternative Conformations and Biology *in DNA Conformation and Transcription*, Springer (2005).

[36] Fuller FB (1978). Decomposition of the linking number of a closed ribbon: a problem from molecular biology. PNAS..

[37] Mirkin, S. M. DNA topology: fundamentals. eLS, *Wiley Online Library* (2001).

[38] Cortini R, Lee DJ, Kornyshev A (2012). Chiral electrostatics breaks the mirror symmetry of DNA supercoiling. J. Phys Condens. Matter.

[39] Varnai P, Timsit Y (2010). Differential stability of DNA crossovers in solution mediated by divalent cations. Nucleic Acids Res..

[40] Timsit Y, Várnai P (2010). Helical Chirality: a Link between Local Interactions and Global Topology in DNA. Plos One.

[41] Japaridze A (2017). Hyperplectonemes: A Higher Order Compact and Dynamic DNA Self-Organization. Nano Lett..

[42] Adamcík J (2002). Effect of bacteria growth temperature on the distribution of supercoiled DNA and its thermal stability. Electrophoresis..

[43] Lifeng Y, Yvasaki IH (2002). Thermal Denaturation of Plasmid DNA Observed by Atomic Force Microscopy. Jpn. J. Appl. Phys..

[44] Zhu M, Lerum MZ, Chen W (2011). How to prepare reproducible, homogeneous, and hydrolytically stable aminosilane-derived layers on silica. Langmuir.

[45] Napoli A (2005). Functional interaction of reverse gyrase with single-strand binding protein of the archaeon Sulfolobus. Nucleic Acids Res..

[46] Hatfield GW, Benham CJ (2002). DNA topology-mediated control of global gene expression in *Escherichia coli*. Annu. Rev. Genet..

[47] Liu Y, Berrido AM, Hua ZC, Tse-Dinh YC, Leng F (2017). Biochemical and biophysical properties of positively supercoiled DNA. Biophys Chem..

[48] Carrasco C, Dillingham MS, Moreno-Herrero F (2014). Single molecule approaches to monitor the recognition and resection of double-stranded DNA breaks during homologous recombination. DNA Repair..

[49] Japaridze A (2017). Spatial confinement induces hairpins in nicked circular DNA. Nucleic Acids Res..

[50] Vvendeskaya IO (2015). Massively Systematic Transcript End Readout (MASTER): Transcription Start Site Selection, Transcriptional Slippage, and Transcript Yields. Mol Cell.

[51] Lang B (2007). High-affinity DNA binding sites for H-NS provide a molecular basis for selective silencing within proteobacterial genomes. Nucleic Acids Res..

[52] Gerganova V (2015). Upstream binding of idling RNA polymerase modulates transcription initiation from a nearby promoter. J Biol Chem..

[53] Schneider R, Travers A, Muskhelishvili G (1997). FIS modulates growth phase-dependent topological transitions of DNA in *Escherichia coli*. Mol Microbiol..

[54] Pivonková H (2010). Selective binding of tumor suppressor p53 protein to topologically constrained DNA: Modulation by intercalative drugs. Biochem Biophys Res Commun..

[55] Suma A, Micheletti C (2017). Pore translocation of knotted DNA rings. Proc Natl Acad Sci USA.

