# Supplementary material for: Structure and Properties of DNA Molecules Over The Full Range of Biologically Relevant Supercoiling States
Source: Sci Rep. 2018 Apr 18;8:6163. doi: 10.1038/s41598-018-24499-5 (PMC5906655; doi:10.1038/s41598-018-24499-5)
Supplement: Supplementary file 1 — Supplementary Figures and legends [file 41598_2018_24499_MOESM1_ESM.pdf]

Supplemetary Information

Structure and Properties of DNA Molecules Over The Full Range of Biologically Relevant Supercoiling States

Paolo Bettotti [1§], Valeria Visone [2§], Lorenzo Lunelli [3,4§], Giuseppe Perugino[2], Maria Ciaramella[2*] and Anna Valenti[2*]

Figure 1S

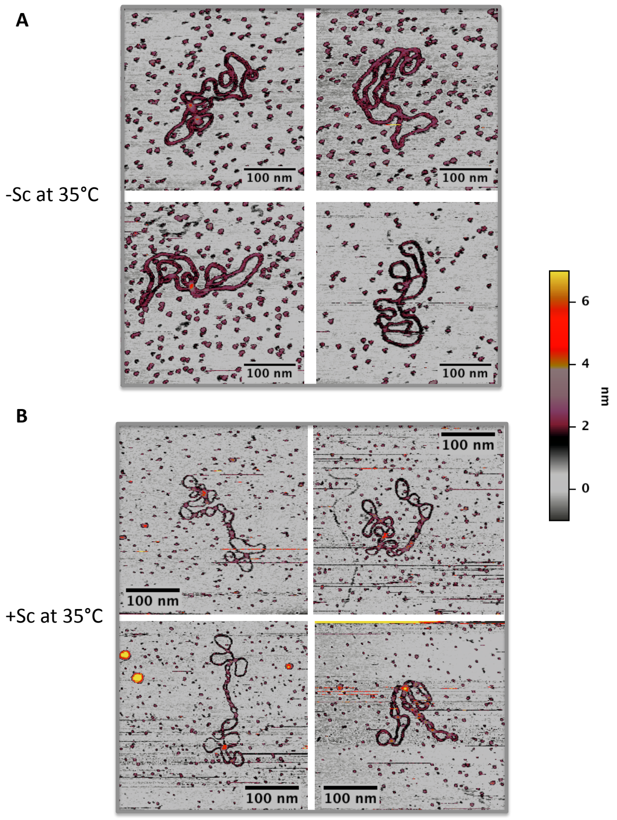

Figure 1S. AFM analysis in solution at 25°C. Representative conformations of –SC (A) and +SC (B) molecules.

Supplemetary Information

Structure and Properties of DNA Molecules Over The Full Range of Biologically Relevant Supercoiling States

Paolo Bettotti [1§], Valeria Visone [2§], Lorenzo Lunelli [3,4§], Giuseppe Perugino[2], Maria Ciaramella[2]* and Anna Valenti[2]*

Figure 2S

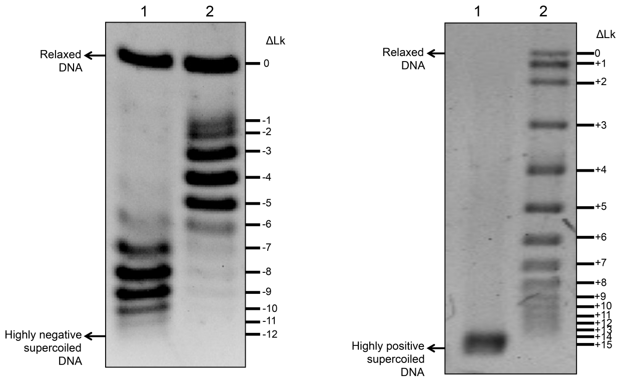

Figure 2S. Determining supercoiling density of –SC and +SC plasmid. A. Gel electrophoresis run in the presence of 1 µg mL-1 chloroquine to determine the superhelical density of negatively supercoiled plasmid. Lane 1, plasmid extracted from E. coli cells grown at 37 °C; lane 2, ladder of negative topoisomers with the indicated linking number. B. Gel electrophoresis without intercalating agents to determine the superhelical density of the end point product of the reverse gyrase; reaction was at 90 °C for 10 min in the presence of 1 mM ATP, with the plasmid substrate shown in lane 1 of fig 1A. Lane 1, positively supercoiled plasmid; lane 2, ladder of positive topoisomers of the indicated ΔLk. Arrows indicate the relaxed and supercoiled forms of the plasmid, respectively. The calculated sigma (σ) values are reported.
